# Wearable, high-density fNIRS and diffuse optical tomography technologies: a perspective

**DOI:** 10.1117/1.NPh.10.2.023513

**Published:** 2023-05-17

**Authors:** Ernesto E. Vidal-Rosas, Alexander von Lühmann, Paola Pinti, Robert J. Cooper

**Affiliations:** a University College London, DOT-HUB, Biomedical Optics Research Laboratory, Department of Medical Physics and Biomedical Engineering, London, United Kingdom; b Gowerlabs Ltd., London, United Kingdom; c Technische Universität Berlin – BIFOLD, Intelligent Biomedical Sensing Lab, Machine Learning Department, Berlin, Germany; d Boston University, Neurophotonics Center, Department of Biomedical Engineering, Boston, Massachusetts, United States; e University of London, Birkbeck College, Centre for Brain and Cognitive Development, London, United Kingdom; f University College London, Department of Medical Physics and Biomedical Engineering, London, United Kingdom

**Keywords:** diffuse optical tomography, functional near-infrared spectroscopy, functional neuroimaging, high-density diffuse optical tomography, wearable

## Abstract

Recent progress in optoelectronics has made wearable and high-density functional near-infrared spectroscopy (fNIRS) and diffuse optical tomography (DOT) technologies possible for the first time. These technologies have the potential to open new fields of real-world neuroscience by enabling functional neuroimaging of the human cortex at a resolution comparable to fMRI in almost any environment and population. In this perspective article, we provide a brief overview of the history and the current status of wearable high-density fNIRS and DOT approaches, discuss the greatest ongoing challenges, and provide our thoughts on the future of this remarkable technology.

## 1 Where We Are Now

Functional near-infrared spectroscopy (fNIRS) and diffuse optical imaging methods have attracted great interest because of their capacity to interrogate cerebral hemodynamics noninvasively while using devices that can be applied in a wide range of experimental circumstances and populations.^1^^,^^2^

The anatomical specificity of fNIRS measurements of human cerebral hemodynamics is a function of the number of source–detector pairs per unit area of the scalp and of the spatial arrangement of those sources and detectors.^3^ Furthermore, fNIRS measurements can be severely influenced by the hemodynamics of extracerebral tissues.^4^ The extension of fNIRS technologies to employ dense arrays of multiple sources and detectors at several different separations (including short separations that are almost exclusively sensitive to the scalp) is commonly referred to as high-density fNIRS or, if three-dimensional (3D) image reconstruction is undertaken, high-density diffuse optical tomography (HD-DOT).^5^^,^^6^ Dense measurements with spatially overlapping sensitivity distributions improve lateral spatial resolution and reduce partial volume blurring, with the availability of measurements at multiple separations providing depth specificity that can effectively minimize extracerebral contamination.^7^

The desire for an increased sampling density has emerged over the last decade and has coincided with the considerable progress in the miniaturization and increased wearability and applicability of fNIRS devices.^8^ Current efforts are now focussed on extending the functionality of fNIRS devices toward fully wearable, wireless, unobtrusive, high-density (HD), and whole-head technologies.^9^^–^^13^

In this perspective article, we describe how the fNIRS field is currently on the cusp of achieving this goal. We also describe the ongoing and future challenges for such technologies and the likely impact that enabling routine, high-quality imaging of cortical haemodynamics in any population and in almost any environment will have on neuroscience.

### 1.1 Definitions

There remain significant variations in how several terms, including “high-density” and “wearable,” are used throughout the fNIRS field. For the sake of clarity, we define the following.

fNIRS: methods that employ near-infrared light to investigate brain function via measurements of changes in hemoglobin concentrations. Although fNIRS has traditionally referred to approaches that collect and examine data on a channel-by-channel basis, the term “fNIRS” is increasingly used as a catch-all for any approach that exploits the diffuse transmission of near-infrared light to measure the human brain.

Diffuse optical tomography (DOT): the use of fNIRS measurements to create 3D images of the optical properties of tissue.

Field-of-view (FOV): the region of tissue to which an fNIRS or DOT array is sensitive.

HD: There is no commonly accepted definition of “high-density” in the context of an NIRS or DOT array yet; here, we consider an array to be “HD” if (a) it contains channels with a range of at least three source–detector distances that span short-separations (<15  mm) and long separations (≥30  mm) and (b) the sensitivity distributions associated with these multidistance channels spatially overlap to provide spatially continuous sampling of the underlying tissue. In practice, this requires an optode density of ∼0.5 to $2\;{\mathrm{cm}}^{-2}$ or more, to be classified as HD.

Wearable: We use this term to describe systems that are sufficiently miniaturized to permit all optoelectronic components to be worn by the subject, typically on the head, with either minimal tethering cabling that provides power and data communications or, in the case of wireless devices, no tethering at all.

## 2 How We Got Here

**Fig. 1:**
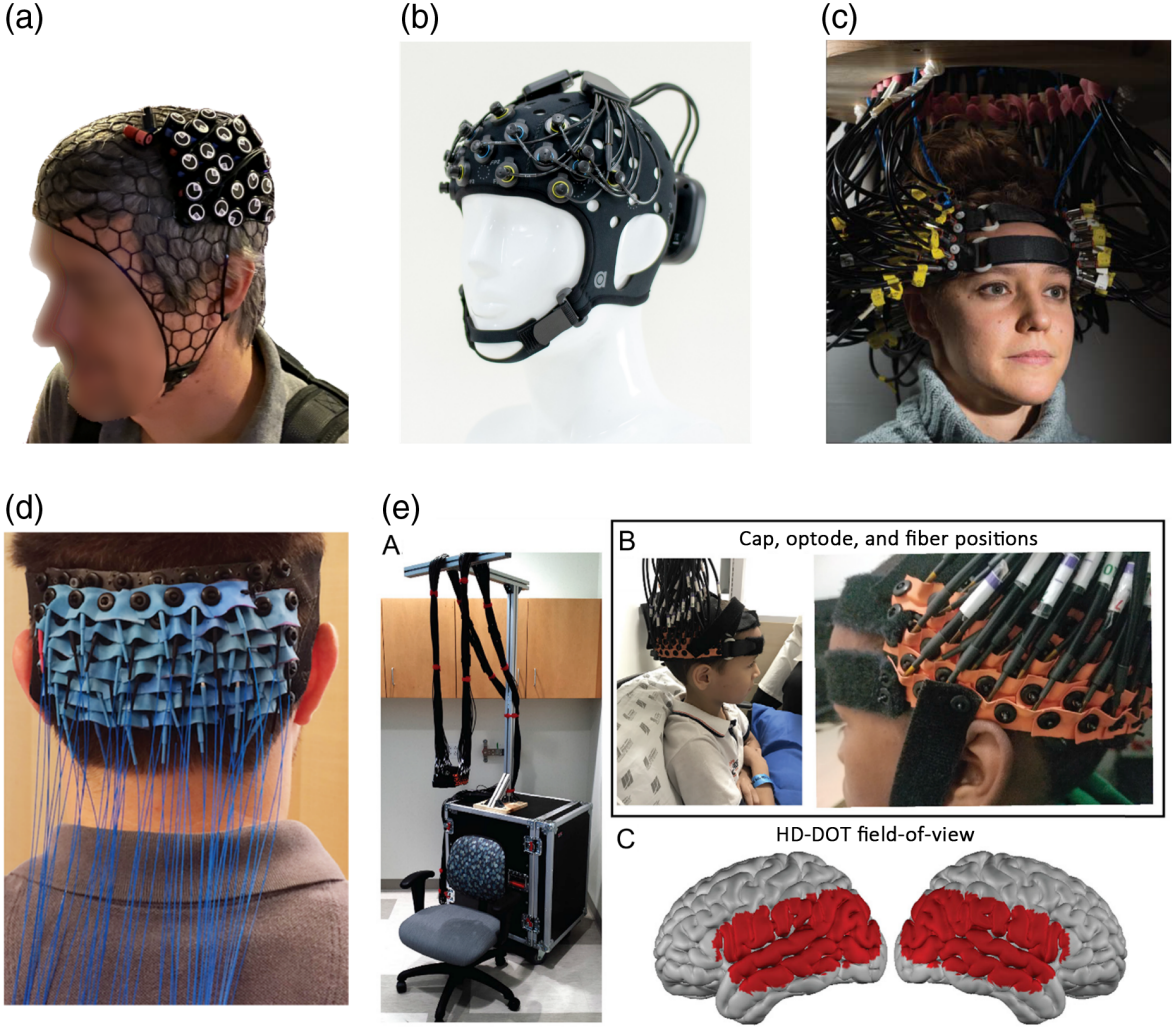
(a) NIRx NIRSport2^14^ (NIRx Medizintechnik GmbH, Germany). Reproduced with permission, courtesy of Bio Optical and Acoustic Spectroscopy Lab, Boston, Massachusetts, USA. (b) Artinis Brite system. Reproduced with permission, courtesy of Artinis Medical Systems, Elst, The Netherlands. (c) In-house continuous-wave (CW) HD-DOT system.^7^ Reproduced with permission, courtesy of Nature Portfolio. (d) Light weight fiber-based CW HD-DOT device.^15^ Reproduced with permission, courtesy of SPIE. (e) Portable fiber-based CW HD-DOT system.^16^ Reproduced with permission, courtesy of Elsevier.

Wearable HD technologies have emerged by building upon the technical developments in wearable fNIRS and fiber-based HD-DOT systems. Wearable fNIRS has made extraordinary progress in the last decade. Detailed reviews of these technologies are already available,^8^ but to summarize, there are now a large range of ultralightweight and wearable fNIRS devices on the market [e.g., the NIRx NIRSport2 (NIRx Medizintechnik GmbH, Germany) and Artinis Brite systems (Artinis Medical Systems, The Netherlands); Figs. 1(a) and 1(b)], several of which are fully wireless and require no components to be worn beyond the head cap itself.

These improvements in fNIRS device wearability and weight have permitted increasingly unrestricted functional neuroimaging, which is reflected in the development of paradigms to study brain function in cognitive tasks that incorporate movement,^17^ exercise,^18^^,^^19^ and dynamic balance.^20^ The rationale behind these studies is to increase ecological validity, that is, the degree to which the task is representative of a real situation.^21^ For instance, fMRI studies that require motor execution resort to mental imagery, i.e., the mental performance of a motor task without actual execution.^22^^,^^23^ However, mental simulation (or surrogate movements) cannot fully capture the execution and corresponding cognitive load of contextualised situations, such as surgeries, teaching, or face-to-face conversations.

Wearable fNIRS has also been used to evaluate expertise and the effect of feedback in laparoscopy procedures,^11^^,^^24^ deception,^25^ stress processing during sleep,^26^ and walking.^12^^,^^27^ A range of studies have also sought to employ wearable fNIRS devices in the study of infants during naturalistic interactions and play.^28^

As wearable fNIRS systems have developed at pace over the last 10 to 15 years, a parallel research effort has been focussed on increasing the spatial resolution and anatomical specificity of fNIRS and DOT approaches through the use of HD optode arrays. Zeff and et al.^6^ introduced HD-DOT technology in 2007. They demonstrated the feasibility of retinotopic functional mapping with a spatial resolution superior to any previous fNIRS or DOT study. Zeff et al.’s in-house continuous-wave HD-DOT device provided partial coverage of the occipital cortex (OCC)with a regular array with a minimum source–detector separation of 13 mm and ∼340 source–detector channels. In 2012, Habermehl et al.^29^ described another HD DOT device, which (uniquely for a continuous wave system) incorporated null source–detector separations measurements. The imaging system was a DYNOT 232 system developed by NIRx medical technologies LLC, and the array consisted of 5×6 fiber bundles that provided ∼900 source–detector pairs. The system demonstrated the capacity to provide somatotopic mapping of the motor cortex with a resolution in the millimeter range, enabling the differentiation of the cortical activation of two fingers on the same hand. These devices both provided only a limited FOV, that is, the arrays only sampled reduced regions of brain tissue.

Further advancement came in 2014 with the introduction of an expanded version of the device employed by Zeff et al. [Fig. 1(c)]. Eggebrecht et al.^7^^,^^30^ demonstrated the mapping of higher-order cognitive functions over the occipital, temporal, and somatomotor cortices; increased connectivity networks; and simultaneous activations in language processing paradigms owing to the larger FOV and improved electronics.

Fiber-based HD systems have generally exploited the high-performance of avalanche photodetectors (APD) to yield the dynamic range and sensitivity necessary for HD measurements, but these devices have generally been restricted to the lab environment. In an effort to develop a fiber-based but more flexible design, Bergonzi et al.^31^ demonstrated the feasibility of using lightweight fiber optics (200-μm multimode fibers) in combination with a high-speed CMOS camera for HD-DOT measurements [Fig. 1(d)]. The authors employed a super-pixel approach to match the performance of larger fiber bundles. However, despite the reduced weight, the fragility of the fiber optics limits the utility of such a design. In a similar vein, Fishell et al.^16^ demonstrated a portable version of a fiber-based HD instrument [Fig. 1(e)]. The authors successfully imaged the hemodynamic response to visual and language stimuli in a pediatric population outside a traditional lab setting.

In recent years, the developments in wearable fNIRS [for instance Fig. 1(b)] and in HD DOT have begun to coalesce toward wearable HD-fNIRS and HD-DOT technologies. This development has required the balance of numerous competing factors. Multidistance measurements and dense sampling are clearly necessary for HD-DOT, and the denser the sampling is, the greater the potential spatial resolution is. But the greater the density of the sampling array is, the greater the electronic and mechanical challenges are. Crucially, detector electronics that provide high dynamic range (many decades) are critical to the measurement of both short and long source–detector separations. Meanwhile, the long list of mechanical and ergonomic requirements includes the need to be lightweight and robust to movement artifacts, to provide effective optode-tissue coupling, and to be flexible enough to conform to the scalp surface.

Fiber-based technologies limit the stability of scalp coupling due to the fiber weight and their vulnerability to movement. A key step in the development of wearable HD methods has therefore been the move to fiberless designs that mount the optical sources, detectors, and even associated acquisition electronics directly onto the scalp. However, mounting extensive optoelectronics onto the scalp poses numerous significant challenges, including issues relating to weight, power consumption, heating, comfort, and (perhaps most significantly) the need to develop manufacturable circuit designs that can still conform to the curved scalp.

An early step into this domain was seen with the Obelab NIRSIT device (Obelab Inc., South Korea). The NIRSIT is a wearable fNIRS device that provides measurements at source–detector separations from 15 to 33 mm with some overlapping spatial sampling. It has the advantage of being fully wireless but is only applicable to the forehead and has, to date, undergone only limited validation.^32^

The advent of modular system architectures has been one of the single most significant steps toward the goal of whole-scalp, HD fNIRS and DOT. Modular, wearable devices employ self-contained optoelectronic modules that typically incorporate one or more source–detector pairs and allow channels to be formed both within and across modules. This approach allows dense networks of channels to be formed while still permitting the system as a whole to conform to the scalp, and it also has advantages in terms of manufacturability, power consumption, and cable minimization.

**Fig. 2:**
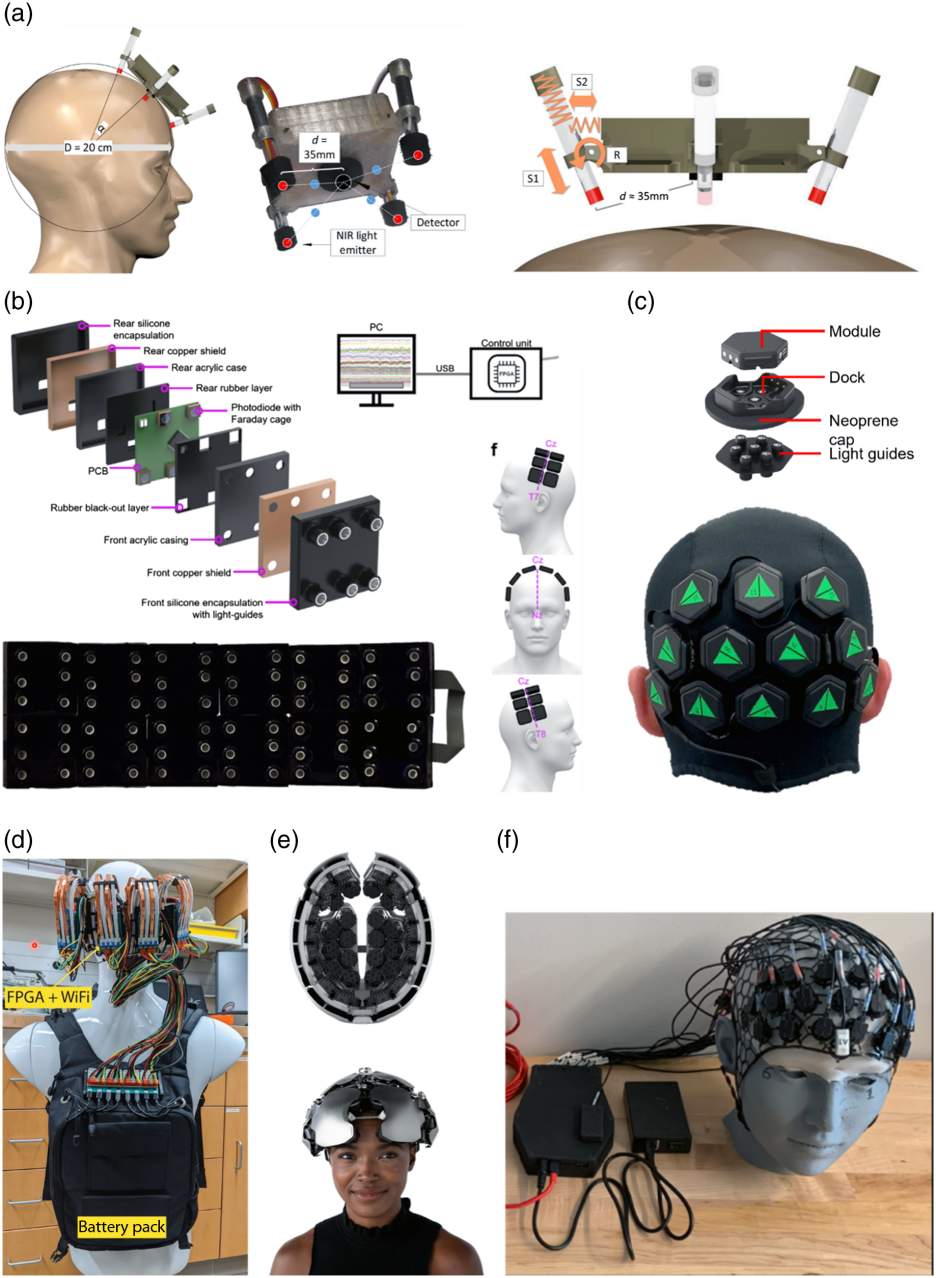
(a) First wearable CW fNIRS system following a modular design.^33^ Reproduced with permission, courtesy of Frontiers Media. (b) μNTS: first wearable modular CW HD-DOT system.^34^^,^^35^ Reproduced with permission, courtesy of OPTICA Publishing Group. (c) LUMO: first commercially available wearable modular CW HD-DOT system.^13^^,^^36^ Reproduced with permission, courtesy of SPIE. (d) In-house CW wearable HD-DOT system.^37^ Reproduced with permission, courtesy of SPIE. (e) Kernel flow: first wearable time-domain DOT system.^38^ Reproduced with permission, courtesy of Kernel, USA. (f) NINJANirs 2021.^39^ Reproduced with permission, courtesy of OPTICA Publishing Group.

The first modular, wearable fNIRS systems were demonstrated by von Lühmann et al.;^33^ although highly innovative, this first generation did not permit channels to be formed across modules [Fig. 2(a)]. A second generation did and even included EEG among other modalities but no HD-DOT.^40^ Chitnis et al.^41^ unveiled the first modular HD-DOT system [Fig. 2(b)]. The authors recognized the advantages in wearability and ergonomics that a modular architecture provides and successfully imaged tomographic haemodynamic changes in the motor cortex. High-quality measurements at source–detector separations from 10 mm to in excess of 50 mm contributed to the resulting high-quality reconstructions. Further developments of the same modular technology showed robust activations over a larger FOV in a walking paradigm,^34^ demonstrating the potential of fiberless modular technology to provide imaging of brain function with extraordinary experimental flexibility.

The first commercially available modular, wearable HD-DOT system was developed by Gowerlabs Ltd. and was first presented at the fNIRS conference in Tokyo in 2018 [Fig. 2(c)]. The LUMO system, which is based on hexagonal sensor modules and provides source–detector separations ranging from 10 up to ∼45  mm, has now been successfully applied across multiple populations, including neonates, infants, and adults.^13^^,^^36^^,^^42^ This system, in the form of a 12-module system applied to the visual cortex, was recently validated in a similar fashion to the original HD-DOT systems using a battery of visual paradigms in adults.^13^ A wider FOV, 24-module implementation has also been applied to examine the repeatability and robustness of functional network mapping in the home setting.^43^

In the last year, several other wearable HD systems have begun to emerge. For example, Agato et al.^37^ introduced a fiberless wearable HD-DOT system that employs pencil-like sources and detectors that brush through hair and provide robust optode coupling [Fig. 2(d)]. The system was validated using retinotopic mapping of the visual cortex and demonstrated comparable performance to the larger fiber-based system.^44^ The recent fNIRS 2022 conference also showcased the increasing number of research teams and companies that are developing new wearable HD devices^45^^–^^48^ or expanding functionality of existing commercially wearable fNIRS imagers toward full HD functionality [Fig. 1(a)].

Although the vast majority of development and miniaturization of fNIRS approaches has been in the continuous-wave domain, i.e., in systems that simply measure changes in optical intensity over time, it is time-domain (TD) diffuse optical technologies that are considered the gold standard of NIRS approaches. This is due to their capacity to resolve absolute values of the scattering and absorption coefficients of tissue. However, the need for high-speed electronics and pulsed lasers has always hindered the development and miniaturization of TD technologies, which have always languished many years behind the development of CW approaches in terms of useability, applicability, and miniaturization.

This status quo was upended in 2021 with the introduction of the first commercially available, wearable time-domain system: kernel flow [Fig. 2(e)]. The flow device takes the same modular architecture approach, with each module consisting of a single, dual-wavelength laser source and six time-resolved detectors, with a minimum source–detector separation of 10 mm.^38^ This technology has great potential to provide improved functional mapping, particularly in terms of quantitation, both within and across individuals. Although significant publications are expected to emerge in the coming months, to date, the demonstration and validation of the flow device, particularly in terms of imaging capability, has been limited.^49^

## 3 Ongoing Challenges

Although enormous progress has been made, there remain several ongoing issues that continue to cause challenges in the development of wearable HD devices, several of which are common to all fNIRS technologies.

### 3.1 Field of View

For a given density of source and detector optodes, the FOV of an fNIRS device will clearly be limited by the total number of source and detector optodes (or modules) that the device can accommodate. Currently, the FOV in commercially available HD systems is mostly limited to a specific brain region, such as the prefrontal cortex (PFC),^11^^,^^19^^,^^24^ the OCC,^13^ or a combination, for example, the bilateral covering of the temporal lobes^36^ or the PFC-OCC,^43^ though this is changing rapidly; at the time of writing, kernel flow is the only system covering the whole head, although not uniformly. Depending on the arrangement, somewhere in the order of 200 source and 200 detector positions are required to provide HD sampling of the entire adult scalp. Achieving routine, full-scalp sampling has long been a goal of DOT device development and is a key reason for the move away from fiber-coupled and individually cabled optode fNIRS and DOT system designs and toward wearable, head-mounted electronics.^9^ However, even for recent, wearable HD and modular devices, scaling up to provide the full FOV remains a challenge. There are, at the time of writing, no whole-scalp, HD, wearable, and wireless devices available to purchase, though this is likely to change in the very near future.

### 3.2 Fully Wireless Operation

Several fNIRS devices that operate without any tethering of the subject have been available for some time now. These devices (which include, e.g., the Artinis Brite and the NIRx NIRSport systems) allow the subject to be fully ambulatory and move freely during recording. However, to date, the systems that are completely free of tethering are limited in terms of channel count, sampling density, and FOV. In contrast, the devices that do offer HD sampling, including of the whole scalp, remain tethered. This is primarily because of the power demands of a device that incorporates ∼200 dual-wavelength optical sources and ∼200 detectors and associated electronic components. Supporting the operation of such an instrument for extended periods with a battery pack small enough to be wearable is challenging, and the development of efficient optoelectronics is critical to achieving this end. Furthermore, the data bandwidth required for fNIRS data transmission scales superlinearly with optode count, meaning that reliable wireless transmission of the data obtained from HD, wearable fNIRS and DOT devices is another significant engineering challenge. Because of this, existing wearable HD devices, such as the Gowerlabs LUMO and kernel flow, are currently only routinely available in formats that use cabling to provide power and data transmission. Although this cabling is minimal, and a dramatic improvement ergonomically over optical fibers, it still tethers the subject to the bench and thus limits the potential applications. However, these issues are likely to be overcome in the very near future: battery operation of the LUMO system has already been demonstrated in limited circumstances^50^ and the NIRSIT (OBELAB, South Korea) has demonstrated that battery-powered, wearable HD sampling devices are feasible (albeit with a limited FOV in this case).

### 3.3 Weight, Ergonomics, and Subject Comfort

The mechanical challenges associated with wearable HD-fNIRS and DOT development include minimizing weight, maximizing wearability, and maximizing subject comfort across all populations, all while ensuring robust optode-tissue coupling, maximizing sampling density, and minimizing cabling.

Fiberless designs have been successful in meeting these challenges in both wearable fNIRS and, more recently, wearable HD devices. The common feature of such devices is that optoelectronic components, including sources and detectors, are mounted directly onto the scalp. The associated drivers and acquisition electronics can be either colocated with the optodes on the head or wired to a nearby acquisition unit. The latter has the obvious cabling weight problems, whereas the former provides an efficient solution if only one transmission cable can deliver power and data transmission. The NIRSPORT (NIRx) and the LUMO (Gowerlabs Ltd.) systems are commercial examples of both approaches, respectively.

However, weight remains a significant challenge, particularly for devices such as kernel flow that incorporate sophisticated high-speed electronics that require thermal management.

The design of the head cap is a crucial component in any wearable HD-fNIRS or DOT system. Cap design presents challenges because of competing requirements, such as optode stability, cap comfort, and ease of installation. The cap design problem is further exacerbated for a whole-head implementation because head size variations add another competing factor in the dilemma of stability versus comfort. A simple solution has been to employ headwear of various sizes to adapt to the different head sizes. The largest asset of wearability is the possibility of unrestricted imaging; therefore, the cap must provide comfort and ergonomically conform to the head curvature while attaining robust attachment to the scalp, even in the presence of movement, for example, in outdoor tasks that require walking. Some problems for whole-head imaging include providing uniform and standardized sampling. The former is a function of the source–detector channel density and has been approached independently by the different research groups and commercial enterprises; however, the latter will require the fNIRS community to agree on adopting a single design or a selection of recommended standardized cap layouts.

### 3.4 Hair

Hair pigmentation and density have a strong influence on the absorption of light at the NIR wavelengths of interest.^51^^,^^52^ Furthermore, the stability of optode coupling to the head is compromised by the presence of significant hair.^53^ The issue of hair becomes more acute as the number and density of optodes increase. Several solutions have been implemented to improve optical coupling, including brush-like optodes,^54^ spring-loaded optodes,^31^ and protruding light guides,^53^ to name a few. However, even when the hair problem can be ameliorated, hair follicles in the scalp are still strong absorbers. Fang et al.^55^ employed Monte Carlo simulations and a realistic head model to study the effect of the density of scalp hair follicles on the calculation of hemoglobin concentrations. They considered hair follicles densities ranging from 1% to 11%, which effectively translates into higher absorbers at the scalp level. By comparing hemoglobin concentrations against a follicle-free head model, their results indicated a miscalculation of ΔHbO and ΔHbR by ∼11% to 130% and ∼26% to 292%, respectively. These results highlight the need of model calibration procedures or improved tissue models to account for hair pigmentation and density. Improved optomechanical designs in combination with high-quality coupling metrics, such as those provided by the software PHOEBE,^56^ will be key to ensuring wearable and HD fNIRS and DOT devices are as effective as possible and suitable for all individuals and populations.

### 3.5 Multimodal Integration

The availability of wearable fNIRS and HD-fNIRS devices enables us to extend neuroscientific investigations beyond the conventional laboratory environment to mobile participants;^8^ however, this evolution brings with it new challenges that must be addressed. These challenges can be divided into two main categories: (1) data deterioration and (2) the interpretation of the observed patterns of cerebral hemodynamics.

In (1), measuring brain activity with wearable fNIRS technologies in unconstrained situations can introduce several additional sources of noise to the recorded data. The likelihood of motion artifacts corrupting the NIRS signals increases when subjects can move freely, such as when walking or speaking.^57^ Body movements also introduce changes in posture and in systemic physiology (e.g., heart rate, respiration, partial pressure of CO_2_, blood pressure, and superficial perfusion) that affect hemodynamics and blood flow in both the extracerebral and cerebral compartments of the head. Such noise components must be taken into account, minimized, and removed for wearable fNIRS methods to be effective.^58^^,^^59^

In (2), extending neuroimaging experiments from highly controlled laboratories to naturistic contexts (e.g., in the outside world, during social interactions, neuromonitoring in hospitals) results in less control over the environment and participants’ spontaneous behavior. These experimental settings become unpredictable; additional events or distractors can add to the planned experimental stimuli and introduce spurious hemodynamic responses beyond those of interest for the experimenter. Therefore, it becomes crucial to develop solutions to disentangle the events of interest from the spurious hemodynamic activity elicited by unwanted stimuli. In addition, we need to consider that our brains do not exist in isolation from our bodies; indeed, the brain is part of the body. Therefore, tracking the brain alone may not be enough to understand what drives the observed brain activity.

Solutions to both of these challenges can potentially be found by the simultaneous acquisition of additional datatypes. The use of motion sensors, such as accelerometers, which are lightweight and miniaturized, can track head movements and help correct fNIRS signals to minimize motion artifacts.^59^^,^^60^ The development of miniaturized and modular HD fNIRS approaches lends itself to the addition of motion sensor chips to the module circuit boards to provide seamless integration of motion sensing and fNIRS neuroimaging.

Through additional noninvasive and/or contactless technologies, such as eye-tracking, motion tracking, or video recordings, participants’ behaviour and body motion can be tracked with high precision and resolution. In addition, brain health and functioning can be assessed, for example, by combining wearable fNIRS approaches with EEG. A systemic physiology augmented functional near-infrared spectroscopy approach^4^^,^^61^ even has the potential to minimize the impact of systemic interference on fNIRS data through the measurement of additional physiological signals, such as heart rate, respiration, blood pressure, and partial pressure of ${\mathrm{CO}}_{2}$.

However, although a core body of research has demonstrated the importance of multimodal measurements in combination with fNIRS approaches,^62^ it is not yet routine. This is primarily because of the lack of meaningful integration between wearable fNIRS technologies and other critical sensing methods, which adds significant technical and experimental burden to those seeking to integrate different instruments themselves. The integration of multimodal data into appropriate data formats, and the subsequent analysis of those data is also a significant bottleneck.

Having signals (NIRS + other sensors) recorded from separate instruments means that each data file has its own output data structure that does not match the data format required by the available software platforms for fNIRS data analysis (i.e., Homer3, NIRS-SPM, NIRS Toolbox, MNE-NIRS, etc.). Therefore, output data need to be manipulated and converted in the required file format, a process that requires substantial programming skills and knowledge of the required input files for the various software. From a hardware perspective, the battery life of the wearable acquisition system needs to be taken into account when the number of connected devices increases.

### 3.6 Data Analysis

In the emerging fNIRS field in the early 2000s, significant research efforts focused on expanding research instruments from single channel to multichannel systems. Research from the late 2000s then switched to developing the analytical and statistical frameworks necessary for extracting robust information from these multichannel systems and addressing their limitations (i.e., motion artifacts, systemic interference). Most of these solutions have been implemented in freeware toolboxes (e.g., Homer2 & 3,^63^ NIRS-SPM,^64^ Brain AnalyzIR/NIRS toolbox,^65^ MNE-NIRS,^66^ NIRSTORM^67^), which helped to establish fNIRS as a popular and user-friendly neuroimaging modality. However, results from these works highlighted the need for even larger systems with extended FOVs and superior spatial resolution.

With the advances in wearable fNIRS and HD-DOT instrumentation in the last decade, the situation has now reversed again, as the availability of high-channel-count devices designed for 3D reconstruction has not yet been matched by appropriate analytical tools to deal with the increased data volumes and analytical complexity. The image reconstruction problem in DOT requires solving a model of light transport in tissue, such as the optical diffusion equation (DE). Routinely, the DE is solved using numerical methods, such as the finite element method (FEM),^68^ which consists of discretizing a computer model of the medium, such as the head and brain, into smaller regions called elements. The DE is modeled for each element and assembled into a system of equations that can be more readily solved to describe the transport of photons in that medium. Although image reconstruction can be readily performed using FEM-based methods provided by, e.g., TOAST++ and NIRFAST^69^^,^^70^ or by modeling light propagation using Monte Carlo simulations,^71^^,^^72^ these routines are not yet fully integrated within other common platforms that can provide for data preprocessing and statistical analyses. Recently, efforts are being made to develop self-contained packages that can provide state-of-the-art preprocessing, 3D image reconstruction, and statistical parametric mapping of HD fNIRS data (e.g., NeuroDOT^73^). As a result, at present, it is common for researchers to have to work across multiple different toolboxes,^74^ each with its specific characteristics and data formats.

## 4 Future

The value of well-controlled laboratory studies of the brain is undeniable. However, it is often less clear how well a given laboratory study represents brain function in a “real world” situation. The external validity of any laboratory experiment is clearly contingent on the specific conditions of that experiment and/or the cognitive process under investigation, but it is our expectation that the highly artificial surroundings and strong constraints on the participant that are associated with fMRI measurements will be gradually complemented and, in some cases, replaced by neuroimaging of naturalistic movement, cognition, and interactions with the environment that are enabled by wearable HD-DOT and fNIRS technologies.^8^^,^^75^^,^^76^ This will continue to improve the ecological validity of neuroimaging results beyond traditional laboratory-based experimentation as neuroimaging under static and artificial laboratory settings is fundamentally valid only under those select conditions and is not necessarily generalizable to naturalistic, dynamic, complex, multisensory, and often unpredictable real-world environments.^77^^,^^78^ Nevertheless, wearable devices are still an asset for laboratory studies, for reasons of portability, speed of use, and especially comfort for the subject, which become more relevant in neonatal and infant studies.

The future use of wearable HD-DOT and fNIRS, potentially complemented and augmented with multimodal measures, will enable new insights but will also require further innovation. On the continuum from conventional “laboratory-based” to “neuroscience of the everyday world” (NEW),^76^ progress will depend on the interaction between improvements in the robustness of brain-activity estimation and the resulting permissible level of experimental freedom and stimulus complexity.

Multimodal wearable HD-DOT and fNIRS will enable novel paradigms to address new scientific questions. For example, measuring the attentional network^79^ or effective connectivity in everyday life.^80^ The current trend of studies employing wearable neuroimaging systems in freely moving subjects indicates a widespread adoption in a variety of topics including cognition during movement, sports, and social interactions.^8^^,^^77^^,^^81^^–^^83^ Wearable systems will continue to open up new possibilities in clinical settings, including in neurodegenerative diseases, such as Parkinson’s disease^84^^,^^85^ and mild cognitive impairment,^86^ and neurodevelopmental conditions, such as autism^87^ and attention-deficit hyperactivity disorder.^88^

**Fig. 3:**
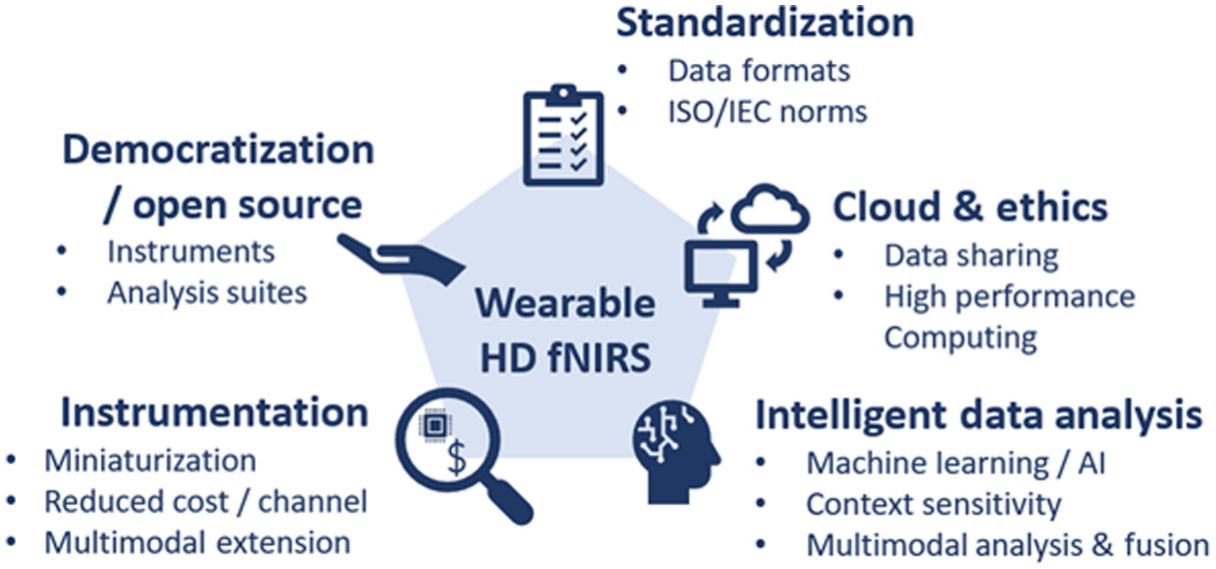
A summary of the key areas of future development of wearable and HD fNIRS technologies and applications.

Below, we provide an outlook on the key domains that will likely be the focus of the next 10 years of wearable HD-DOT and fNIRS development and application. As depicted in Fig. 3, these domains will include: (1) instrumentation, (2) democratization, (3) standardization, (4) cloud sharing and high-performance computing, and (5) intelligent data analysis.

### 4.1 Instrumentation

As we describe above, the fNIRS field is on the cusp of achieving routine, wearable, wireless, whole-scalp HD DOT. We anticipate that, within the next few years, several devices meeting this specification will become commercially available. This will represent an enormous achievement and will be the result of a collective effort across multiple labs, companies, and domains. The instrumentation will likely continue to progress in terms of dynamic range and sensitivity, making high-quality data collection progressively easier with time and helping to offset issues related to the absorbance of hair. These efforts will be supported by the adoption of more sophisticated detection technologies, such as silicon photon multipliers, which outperform typical photodiodes and APDs.^89^^,^^90^ The continued advancement and miniaturization of TD-NIRS technologies, which will likely continue to be pioneered at kernel, will also be a key area of technical development in the coming years.

The increasing availability of multiwavelength optical sources is likely to be exploited to provide wearable fNIRS and HD-DOT devices that are sensitive to chromophores in addition to the hemoglobins, such as cytochrome-c-oxidase.^91^ This can permit the simultaneous measurement and imaging of cortical tissue oxygenation and metabolism,^92^^,^^93^ which has significant clinical and neuroscientific potential.

In the medium to long term, the most dramatic step forward may well be in the development of devices that measure not only changes in cerebral haemoglobin concentrations but also cerebral blood flow, for example, through the combination of fNIRS methods with diffuse correlation spectroscopy^94^ or speckle contrast optical spectroscopy/tomography.^95^^,^^96^ This will again involve different areas of development (in this case speckle-based optical sensing approaches and wearable HD-fNIRS and DOT approaches) coalescing toward a common goal. A path toward a wearable device that yields whole-cortex maps of absolute hemoglobin concentrations, oxygenation, flow, and metabolism is just beginning to emerge.

### 4.2 Democratization and Open Source Development

In recent years, there has been an increased interest in making neuroimaging more broadly accessible. Initiatives include opennirs,^33^^,^^97^ openfnirs,^98^ NinjaNIRS^39^^,^^99^ [Fig. 2(f)], Open FlexNIRS,^100^ and DIY-fNIRS,^101^ which have led this effort in the fNIRS community. The impact of these initiatives can be far-reaching and will particularly benefit researchers and clinicians in less economically developed countries. Studies using fNIRS in rural and resource-poor areas in Gambia, Bangladesh, and Côte d’Ivoire have already provided great insights into the environmental effects on neurodevelopment and learning.^102^^–^^104^ The open initiatives for fNIRS devices are critical to promoting the democratization of fNIRS across the general population. Current initiatives mostly focus on fNIRS devices; however, future developments will no-doubt expand into wearable and HD devices. This trend has already begun with the NinjaNIRS program,^39^ which is an initiative to produce a cost-effective and accessible platform to perform fNIRS studies in naturalistic environments. The system is wearable, scalable, modular, and fiberless to provide robustness to motion artifacts while simultaneously allowing for unrestricted brain monitoring. The potential of wearable HD-fNIRS and DOT devices to yield high-quality functional imaging of the brain at low cost and provide a genuine alternative to fMRI in almost any environment could (and should) have a huge impact on the prevalence of neuroimaging and neuroscience globally. Programs that seek to ensure open access to these technologies will be critical to achieving this goal.

### 4.3 Standardization

Standardization is essential to enabling easy sharing of data and ensuring comparable acquisition device performance. Future datasets will be recorded with wearable HD-DOT instruments built and tested according to international norms (see, for example, the developing fNIRS device standard IEC 80601-2-71) and will be stored using established, open-source international data recording formats [e.g., the shared near-infrared format, snirf^105^ available in a GitHub repository at: https://github.com/fNIRS/snirf, the Brain Imaging Data Structure (BIDS),^106^ NeuroJSON^107^]. The signal processing streams will follow established standards, so the analysis of shared data can be repeated anywhere by anyone. None of these solutions exists in full as yet, but there is a declared interest in promoting the standardization of fNIRS procedures; one example is the recent appearance of the recommended guidelines of fNIRS publications.^62^

In addition to the required standardization of data formats and processing, there are key aspects of hardware standardization that should be considered in the coming years. There is currently no standard layout for optode positioning, making it difficult to compare and interpret results from different manufacturers and groups. It is recognized that consensus equivalent to the EEG 10-20 format is unlikely; however, some intermediate alternatives can be agreed upon, and HD-DOT can help address the issue. In a manner distinct from fNIRS, which can only sparsely cover cortical areas of interest, HD-DOT can achieve uniform coverage by minimizing the interoptode or intermodule gaps, and this uniform coverage can be obtained through any number of optode arrangements. Therefore, quantified standards in the sensitivity distributions that HD optode arrays can yield, or at least standard ways of calculating and presenting absolute, quantified sensitivity maps for HD-DOT devices, would be a big step forward. Another approach would be to establish a set of recommended array geometries and shapes (hexagonal, triangular, etc.) that all meet established thresholds in absolute sensitivity and uniformity, while still allowing for variation in mechanical and optoelectronic design, including for both modular and free-optode arrays.

### 4.4 Cloud Computing and Ethical Considerations

There are two major opportunities in moving fNIRS to the cloud. The first will support machine learning and AI applications, which offer great potential for fNIRS analysis approaches. At the same time, the size and availability of datasets are both increasing. This is as a direct consequence of the increasing prevalence of higher channel count (HD) and data rich (e.g., TD fNIRS) devices. One outcome of larger datasets is that even established analysis approaches can become prohibitively computationally expensive and untenable using typical computing resources. This problem is compounded by the increasingly sophisticated analyses that richer datasets can enable. Cloud computing can provide the necessary processing power and memory space to overcome these challenges and is already available and extensively used across other domains in data science and machine learning.

The second major application space for cloud computing approaches relates to file format standardization and open access to experimental datasets, which will enable the research community to develop powerful new tools for sharing and reproducing research results. Not only can standardized data be shared and validated centrally, but when researchers also share their processing streams, data can also be processed on the cloud, and the results can be reproduced or expanded upon by any researcher with minimal effort. A recent effort by Ref. 98 is working toward such a solution in Ref. 108; the following functionality is available or currently in implementation:

Users can (1) upload snirf files from which (2) BIDS meta-data files are extracted and a compliance check and report are generated identifying any required meta-data that is missing. The user is then directed to where to enter that meta-data. Then, (3) a data quality report is generated using MNE NIRS.^109^ In the future, researchers will be encouraged to also provide their processing and configuration files (e.g., Homer3 proc streams) with the datasets. These data repositories can then be shared with the community for download. Analysis can then be done and repeated 4A) offline or 4B) in the future, online on the cloud by processing scripts on BIDS-compliant datasets that have been shared by users together with the datasets. Cloud-based analysis can help with the reproducibility of results and the acceleration of new processing approaches and collaborative analysis on the same data in the community. In the future, the goal is to enable the construction and running of processing pipelines fully on the web-based interface on the cloud.

One major issue for future wearable neuroimaging using fNIRS approaches, particularly if data are centralized and published through cloud computing approaches, will be the ethical considerations associated with such data. For example, there is the risk that combined multimodality datasets (eye-tracking, video, movement tracking, GPS, etc.) may inadvertently disclose a participant’s identity. The unique and individual-specific nature of functional neuroimaging data and the as-yet unknown power of large and long-duration functional imaging datasets to provide information about an individual’s health and personal characteristics poses the risk that such datasets may betray information about the health status, personal preferences, or inclinations of an individual. Furthermore, because brain data can be revisited for novel analysis, the user might be at risk of consenting to data handling for processes that have not even been conceived. These concerns will likely have to be discussed in panels or topical meetings for the benefit of the fNIRS community.

### 4.5 Intelligent Data Analyses

The use of fNIRS systems in more complex, dynamic, and multisensory environments further increases the need for an improved understanding of the systemic physiological confounds present in the signal and for robust approaches to separate the weak neural information from strong physiological contamination and other artifacts. HD-DOT helps this process by improving brain versus scalp discrimination. Multimodal measures can also support this effort by aiding in the identification and separation of non-neuronal components from the fNIRS signal. The analysis workflow for wearable HD-fNIRS and DOT has to ultimately accomplish several things: (1) the robust removal of nuisance signals from HD-DOT and fNIRS datasets; (2) the automatic annotation of and adaptation to real-world stimuli; and (3) the joint analysis of multimodal HD-DOT and behavioral and additional (e.g., EEG, motion sensing) data. This is an extraordinary and complex challenge and one that is likely to only be overcome through the application of intelligent data analyses that leverage machine learning and AI approaches and multimodal datatypes to automatically disentangle the effects of the complex real-world environment on our measures of cortical hemodynamics.

## 5 Conclusions

This perspective article provides a concise view of the past, present, and future of wearable fNIRS and HD-DOT devices. The fNIRS research field and our numerous commercial partners have responded with extraordinary success to the demands for greater applicability and spatial resolution by making ultra-lightweight and wearable HD fNIRS and DOT systems a reality. This challenge has involved finding a balance between many competing factors: realizing a lightweight, wearable, and comfortable system that simultaneously provides robust optical coupling, high sampling density and with minimal (or nonexistent) tethering of the subject. Although many of the associated engineering difficulties have been addressed, there is still a need for improved optoelectronics, high-bandwidth wireless data streaming, reduced power consumption, and improved solutions to the issue of providing good optical coupling for all hair types. Standardizing processing workflows and data handling procedures is also becoming increasingly critical. The fNIRS community has demonstrated a genuine desire and enthusiasm to address all of these issues. In summary, wearable fNIRS and HD-DOT technologies have made incredible advances in the last few years, and the stage is set to exploit this outstanding technology to address both old research questions that have to-date been impossible to answer and new research questions that have yet to be imagined.
